# Supplementary material for: Transcriptomics-Based Identification of Genes Related to Tapetum Degradation and Microspore Development in Lily
Source: Genes (Basel). 2022 Feb 17;13(2):366. doi: 10.3390/genes13020366 (PMC8872214; doi:10.3390/genes13020366)
Supplement: Supplementary file 1 [file genes-13-00366-s001.zip › genes-1589677 -supplementary Table S1 and Figures.pdf]

Table S1. Primers used in this study.

Genes		Sequence(5'to 3')	Fragment size(bp)
MYB80	Forward primer	CACTCTGTCGTCGGAAAC	154
	Reverse primer	TAAGGTGGGAGAAAGGCT	
AMS	Forward primer	CAAACGGCTGGGACTACT	225
	Reverse primer	CATGTATCCCGATTGAGG	
GSL5	Forward primer	GGCATTAGTAAGGCATCC	138
	Reverse primer	CTGGTTCAGTCCAACATCA	
TDF1	Forward primer	GTCAGGACCCATCACAAC	194
	Reverse primer	CAAGGAAATCACTCCACTC	
SERK2-like	Forward primer	TCTACGCCTTTGGTGTCC	143
	Reverse primer	AGCAGCCTCCTGTTTTGA	
AGAMOUS	Forward primer	TCATCTTCCTCCTCCGTT	238
	Reverse primer	CCTTCTTCAGCAGTCCATT	
LLA-115	Forward primer	GGGGAATGACGACACCTT	150
	Reverse primer	CACCACTGAATCCAAACCT	
NOZZLE	Forward primer	AGACGGAGGTGACGGAGTA	117
	Reverse primer	CAACAAAATGAGGAGACGGT	
GLOX1	Forward primer	ATTCAGTCTGGCGGCTAC	194
	Reverse primer	GATTTGGGGATGAACTCGTA	
ARF17	Forward primer	TTTCCCAAAGGTGCTCAAC	118
	Reverse primer	CTGGAATGGCAGATAAAGAG	
MYB24	Forward primer	CAACCACCTTCTCCTTCA	135
	Reverse primer	CTCCGCTTCTTCTCCACC	
FPA	Forward primer	AGCCCTTTAGCCATTCAC	178
	Reverse primer	TCTGCTTCAGCCTCTTCC	
CYP704B1	Forward primer	CAAACGCCACTGTCACGC	233
	Reverse primer	GTCTTGTTCCTCGCCCAC	
chalcone synthase (CHS)	Forward primer	GGGCTAACATTCCACCTA	116
	Reverse primer	CAGAAAATCGAGTTCCAGTC	
chalcone isomerase (CHI)	Forward primer	TTTTCCTGCTGCCACTCC	124
	Reverse primer	GCTCCCACCCAAATACCA	
cysteine proteinase (CYS)	Forward primer	CGTTGAAGGGGAATCATAAG	162
	Reverse primer	CAGTTGCCTGTTTGTAAGC	
18S	Forward primer	AGTTGGTGGAGCGATTTGTCT	
	Reverse primer	CCTGTTATTGCCTCAAACTTCC	

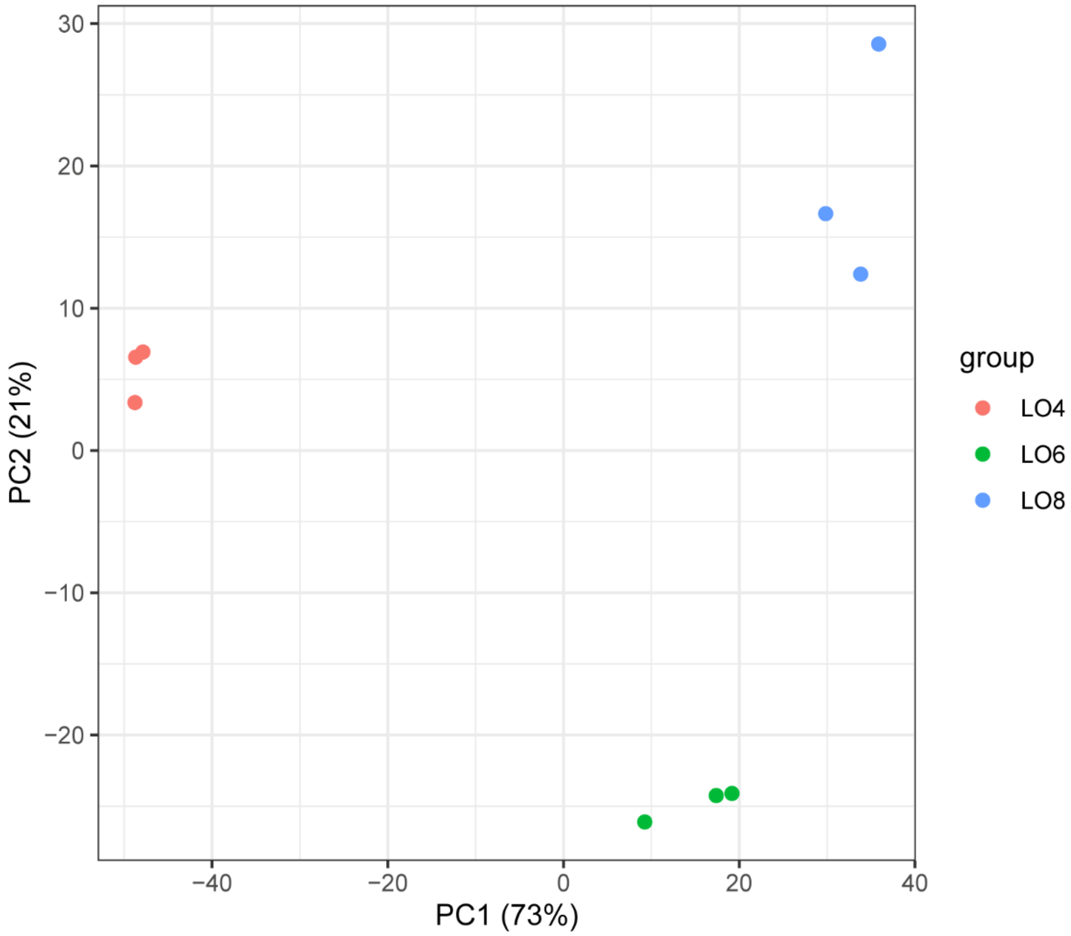

Figure S1. Biological replicates of three anther stages showed by the first two PCs which explained over 94% of variance among samples.

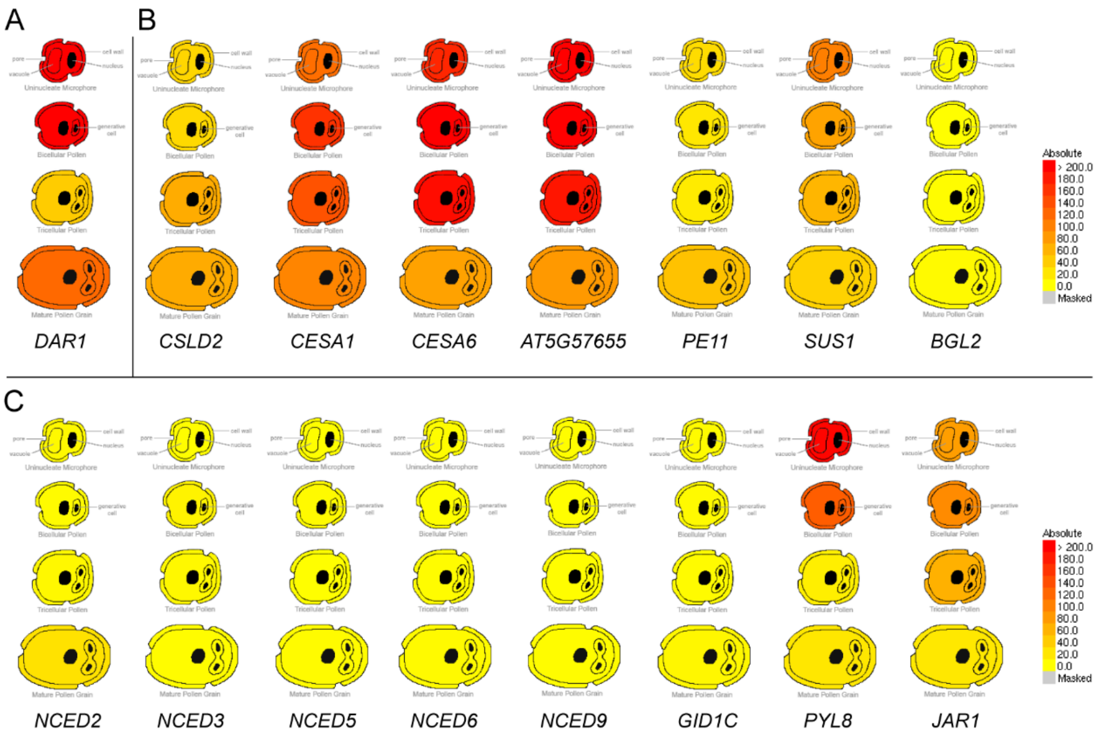

Figure S2. The 'eFP-seq' browser of genes related to the tapetum degradation and microspore development. The color scale from yellow to red represents expression levels from low to high.
